# Supplementary material for: A Humanized Yeast Phenomic Model of Deoxycytidine Kinase to Predict Genetic Buffering of Nucleoside Analog Cytotoxicity
Source: Genes (Basel). 2019 Sep 30;10(10):770. doi: 10.3390/genes10100770 (PMC6826991; doi:10.3390/genes10100770)
Supplement: Supplementary file 1 [file genes-10-00770-s001.zip › Additional_Files/Additional_File7_GOTermHeatmaps.zip!/Additional_File7_GOTermHeatmaps/transport_vesicle.pdf]

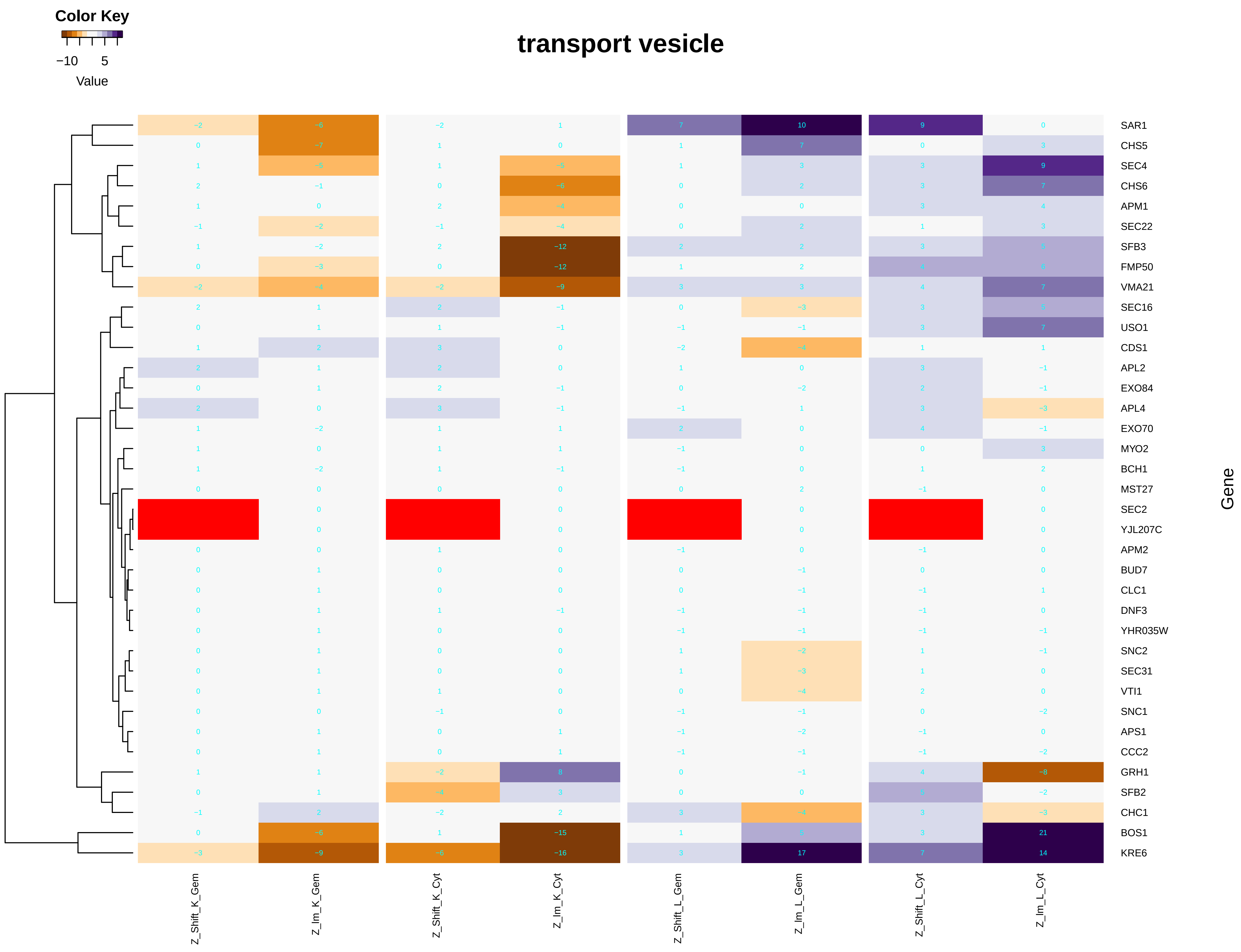
Color Key
−10
5
Value
transport vesicle
SAR1
CHS5
SEC4
CHS6
APM1
SEC22
SFB3
FMP50
VMA21
SEC16
USO1
CDS1
APL2
EXO84
APL4
EXO70
MYO2
BCH1
MST27
SEC2
YJL207C
APM2
BUD7
CLC1
DNF3
YHR035W
SNC2
SEC31
VTI1
SNC1
APS1
CCC2
GRH1
SFB2
CHC1
BOS1
KRE6
Gene
Z_Shift_K_Gem
Z_Im_K_Gem
Z_Shift_K_Cyt
Z_Im_K_Cyt
Z_Shift_L_Gem
Z_Im_L_Gem
Z_Shift_L_Cyt
Z_Im_L_Cyt

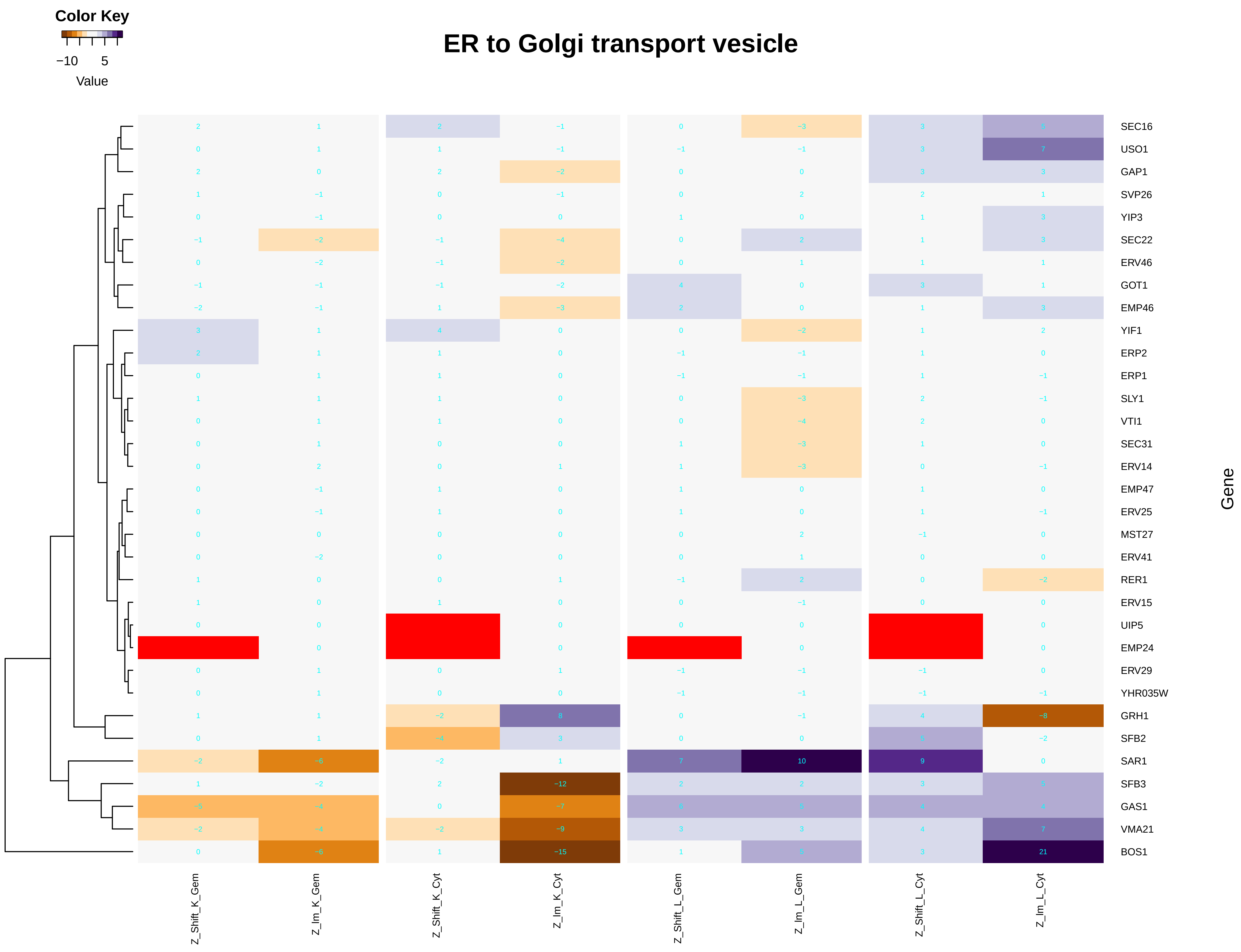
ER to Golgi transport vesicle
Color Key
−10
5
Value
Gene
SEC16
USO1
GAP1
SVP26
YIP3
SEC22
ERV46
GOT1
EMP46
YIF1
ERP2
ERP1
SLY1
VTI1
SEC31
ERV14
EMP47
ERV25
MST27
ERV41
RER1
ERV15
UIP5
EMP24
ERV29
YHR035W
GRH1
SFB2
SAR1
SFB3
GAS1
VMA21
BOS1
Z_Shift_K_Gem
Z_Im_K_Gem
Z_Shift_K_Cyt
Z_Im_K_Cyt
Z_Shift_L_Gem
Z_Im_L_Gem
Z_Shift_L_Cyt
Z_Im_L_Cyt

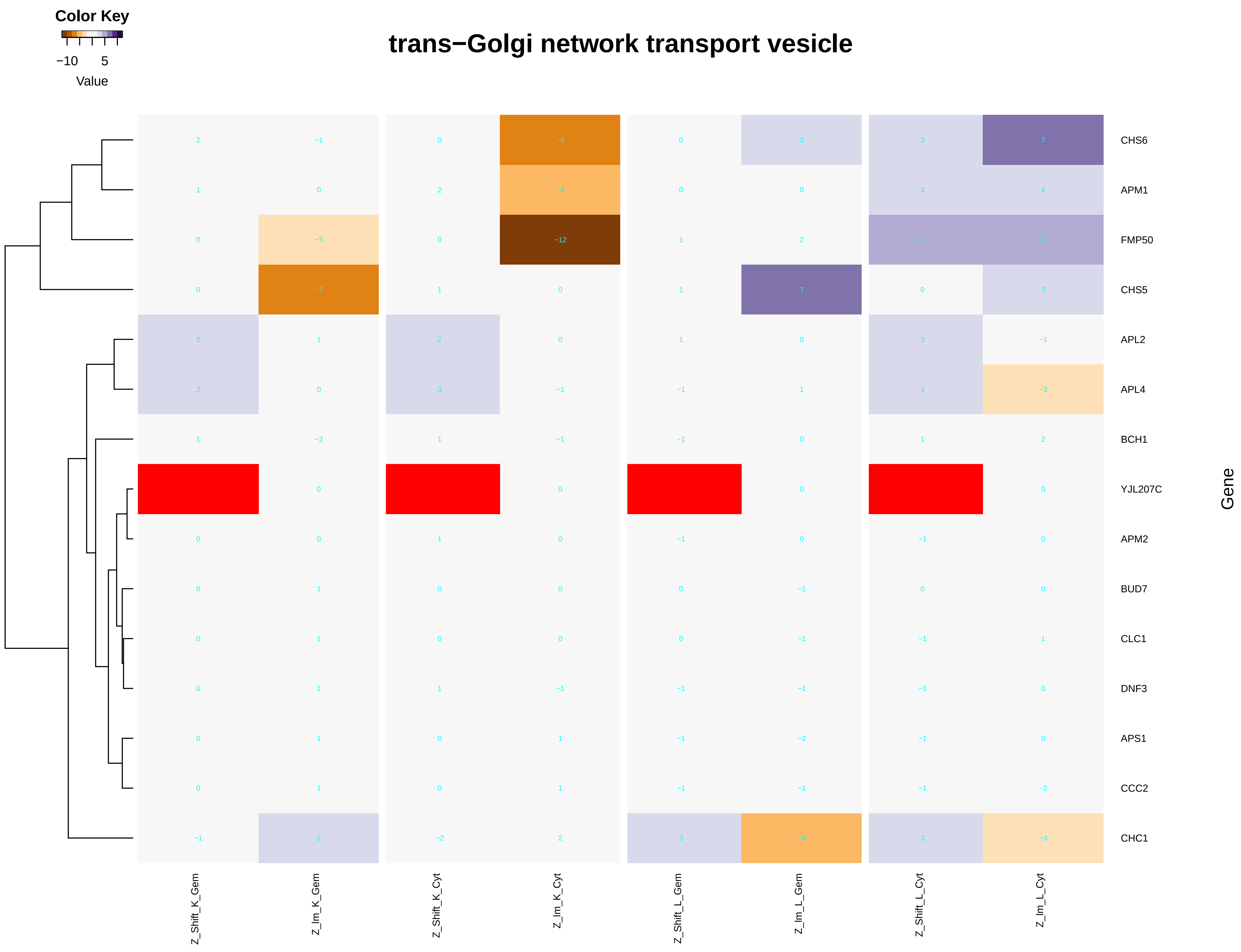

Color Key
−10
5
Value
trans−Golgi network transport vesicle
CHS6
APM1
FMP50
CHS5
APL2
APL4
BCH1
YJL207C
APM2
BUD7
CLC1
DNF3
APS1
CCC2
CHC1
Gene
Z_Shift_K_Gem
Z_Im_K_Gem
Z_Shift_K_Cyt
Z_Im_K_Cyt
Z_Shift_L_Gem
Z_Im_L_Gem
Z_Shift_L_Cyt
Z_Im_L_Cyt
